# Cd-driven surface reconstruction and photodynamics in gold nanoclusters†

**DOI:** 10.1039/d0sc05163b

**Published:** 2021-01-05

**Authors:** Xu Liu, Guo Yao, Xinglian Cheng, Jiayu Xu, Xiao Cai, Weigang Hu, Wen Wu Xu, Chunfeng Zhang, Yan Zhu

**Affiliations:** School of Chemistry and Chemical Engineering, Nanjing University Nanjing 210093 China zhuyan@nju.edu.cn; School of Physics, Nanjing University Nanjing 210093 China; School of Physical Science and Technology, Ningbo University Ningbo 315211 China xuwenwu@nbu.edu.cn

## Abstract

With atomically precise gold nanoclusters acting as a starting unit, substituting one or more gold atoms of the nanocluster with other metals has become an effective strategy to create metal synergy for improving catalytic performances and other properties. However, so far detailed insight into how to design the gold-based nanoclusters to optimize the synergy is still lacking, as atomic-level exchange between the surface-gold (or core-gold) and the incoming heteroatoms is quite challenging without changing other parts. Here we report a Cd-driven reconstruction of Au_44_(DMBT)_28_ (DMBT = 3,5-dimethylbenzenethiol), in which four Au_2_(DMBT)_3_ staples are precisely replaced by two Au_5_Cd_2_(DMBT)_12_ staples to form Au_38_Cd_4_(DMBT)_30_ with the face-centered cubic inner core retained. With the dual modifications of the surface and electronic structure, the Au_38_Cd_4_(DMBT)_30_ nanocluster exhibits distinct excitonic behaviors and superior photocatalytic performances compared to the parent Au_44_(DMBT)_28_ nanocluster.

## Abstract 2

With dual modifications of the surface and electronic structure, Au_38_Cd_4_(DMBT)_30_ exhibits distinct excitonic behaviors and photocatalytic performances compared to Au_44_(DMBT)_28_.

## Graphical abstract

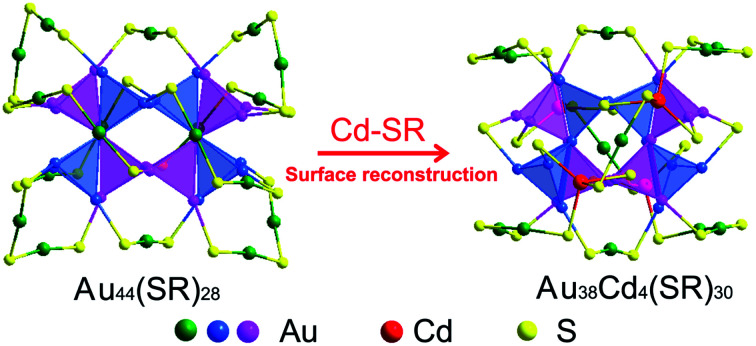

## Introduction

Metal synergy is of paramount importance as the rationale to modulate the intrinsic properties of metal nanoparticles.^1,2^ However, the precise synergistic interaction in an intermetallic nanoparticle has so far been elusive, due to the challenges in determining the atomic-level arrangement of the metal heteroatoms in the nanoparticle. Atomically precise metal nanoparticles (often called nanoclusters) lead to unprecedented opportunities in signalling clear directions to exploit the cooperativity between the two metal elements within a single nanocluster.^3^ Thiolate-protected gold nanoclusters, Au_*n*_(SR)_*m*_, where *n* is the number of gold atoms and *m* is the number of thiolate ligands, SR, have gained momentum over the past few years as an exciting area and have opened up new horizons in precise tailoring of the composition and structure to control the physicochemical properties.^4–12^ The Au_*n*_(SR)_*m*_ nanoclusters are typically configured with an inner gold core (or kernel) and various surface motifs, in which the motifs containing both gold and thiolate resemble staples. Both the gold core and the surface motifs can contribute to the physicochemical properties such as the optical and electronic properties, as well as catalysis.^13–18^ It has been recognized that substituting one or more gold atoms in either the core or the motifs with other metals can tune the overall performances of the parent nanoclusters.^19–26^ Therefore, it has become possible to access the previously inaccessible metal synergy in the bimetallic nanoclusters with atomic-precision.

Among the gold-based bimetal nanoclusters, cadmium-containing bimetal clusters provide synergistic strategies to adjust the electronic structures and further modulate the physicochemical properties in the clusters, since Cd has one more valence electron than Au.^21,26,27^ Cd introduction usually causes surface reconstruction of gold nanoclusters. For example, Au_19_Cd_2_(SR)_16_ was obtained through the substitution of two neighboring surface Au atoms with one Cd with the cuboctahedral Au_13_ unchanged.^26^ Au_19_Cd_3_(SR)_18_ was formed by retaining the icosahedral Au_13_ core but only changing the surface of Au_25_(SR)_18_.^27^ However, the surface reconstruction strategy remains challenging and no examples of bimetal clusters formed without breaking the face-centered cubic (fcc) core of the parent gold clusters have been documented, which might thus impede gaining a higher understanding of how to tailor the surface structure of gold-based nanoclusters and accordingly optimize their synergy.

Herein, we report our success in synthesizing a Au_38_Cd_4_(DMBT)_30_ (DMBT = 3,5-dimethylbenzenethiol) nanocluster that is obtained from the surface reconstruction of Au_44_(DMBT)_28_ induced by Cd^2+^. The fcc Au_26_ kernel originating from Au_44_(DMBT)_28_, which is assembled from Au_4_ tetrahedra, is retained in the Au_38_Cd_4_(DMBT)_30_. The Au_38_Cd_4_(DMBT)_30_ nanocluster exhibits distinctly different excited-state dynamics from Au_44_(DMBT)_28_. More interestingly, the Au_38_Cd_4_(DMBT)_30_ nanocluster as a photocatalyst shows better visible light-driven catalytic activity than the parent Au_44_(DMBT)_28_ catalyst.

## Results and discussion

**Fig. 1:**
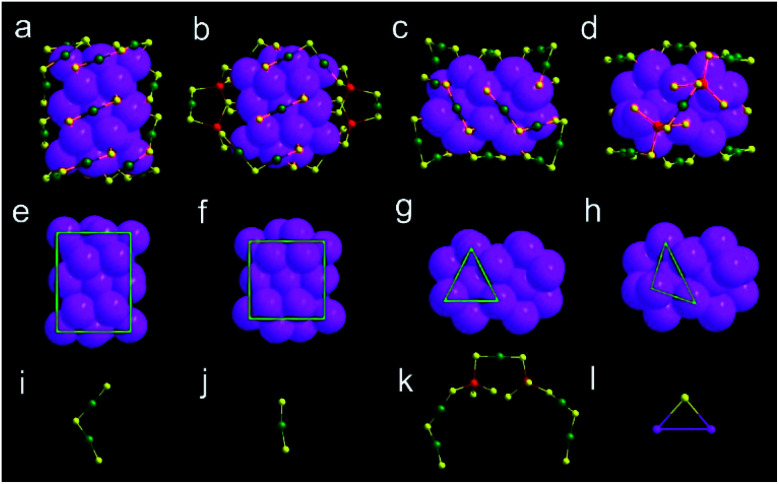
Structural analysis of Au_44_(DMBT)_28_ and Au_38_Cd_4_(DMBT)_30_ nanoclusters in the space-filling mode. Total structures of (a) Au_44_(DMBT)_28_ and (b) Au_38_Cd_4_(DMBT)_30_ viewed from the front. Total structures of (c) Au_44_(DMBT)_28_ and (d) Au_38_Cd_4_(DMBT)_30_ viewed from the side. Au_26_ kernels of (e) Au_44_(DMBT)_28_ and (f) Au_38_Cd_4_(DMBT)_30_ viewed from the front. Au_26_ kernels of (g) Au_44_(DMBT)_28_ and (h) Au_38_Cd_4_(DMBT)_30_ viewed from the side. Note that green frames show kernel distortions. Various motifs of the two nanoclusters: (i) Au_2_(SR)_3_; (j) Au(SR)_2_; (k) Au_5_Cd_2_(SR)_12_; (l) SR; color codes: magenta/green = Au, yellow = S, red = Cd. C and H atoms are omitted for clarity.

X-ray crystallography analysis shows that the parent Au_44_(DMBT)_28_ nanocluster is composed of an Au_26_ kernel, six Au_2_(SR)_3_ and two Au(SR)_2_ staples (Fig. 1a, c and Table S1†). The formula of Au_44_(DMBT)_28_ is further confirmed by electrospray ionization mass spectroscopy (ESI-MS, Fig. S1a†). The structural framework of Au_44_(DMBT)_28_ is identical to that of the reported Au_44_(TBBT)_28_ (TBBT = 4-*tert*-butylbenzenethiol) (Fig. S2†),^28^ both of which can be assembled into the layered structures (Fig. S3–S5†). Notably, a significant difference is observed in the layer's interior, where all the molecules of Au_44_(TBBT)_28_ in the layer (marked with the same color, Fig. S3†) are packed along the same direction, while Au_44_(DMBT)_28_ molecules are arranged in different directions (Fig. S5†). Such a difference may be ascribed to the different steric hindrance between TBBT and DMBT. The UV-vis-NIR spectra of the two Au_44_(SR)_28_ nanoclusters show only small deviations. As shown in Fig. S6,† the prominent peak at 380 nm for Au_44_(TBBT)_28_ is slightly red-shifted to 388 nm for Au_44_(DMBT)_28_, and the broad peaks at 650 and 725 nm become apparent when TBBT is replaced by DMBT.

**Fig. 2:**
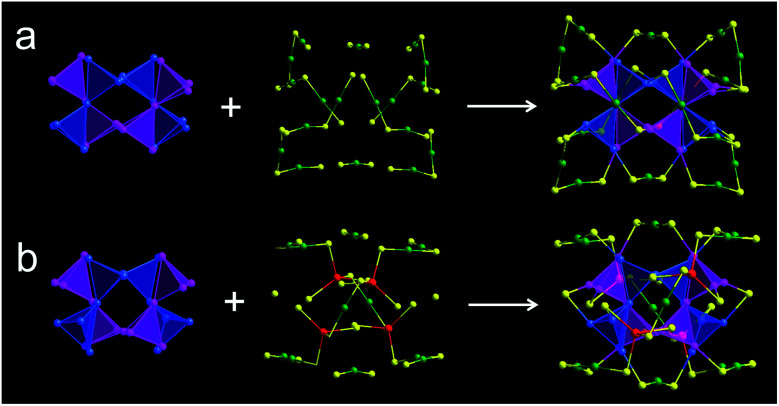
Structural anatomy of (a) Au_44_(DMBT)_28_ and (b) Au_38_Cd_4_(DMBT)_30_ with the fcc Au_26_ kernels assembled from Au_4_ building blocks. Color codes: blue/magenta/green = Au, yellow = S, red = Cd. C and H atoms are omitted for clarity.

With Au_44_(DMBT)_28_ as a starting unit, a Cd-doped nanocluster was further synthesized *via* an ion-exchange strategy. From ESI-MS data (Fig. S1b†), the prominent peak at 6025.43 *m*/*z* with a +2 charge is assigned to Au_38_Cd_4_(DMBT)_30_ (theoretical value: 6025.48 *m*/*z*), which is further confirmed by the excellent match between experimental and calculated isotopic patterns (inset of Fig. S1b†). Single crystallography analysis reveals that Au_38_Cd_4_(DMBT)_30_ contains a 26-Au-atom kernel, two Au_5_Cd_2_(SR)_12_ staples, two Au(SR)_2_ staples and two bridging SR ligands, as shown in Fig. 1b, d, and Table S2.† Note that the retained kernel of Au_38_Cd_4_(DMBT)_30_ experiences a slight distortion from “slender” to “stocky” in comparison with that of the parent Au_44_(DMBT)_28_ (Fig. 1e–h). Further analysis shows that the Au_26_ kernel in Au_38_Cd_4_(DMBT)_30_ can be viewed as the assembly of tetrahedral Au_4_ units in a double-helical mode, as well as that in Au_44_(DMBT)_28_ (Fig. 2). Furthermore, the two nanoclusters have almost identical distances between neighboring Au_4_ units, which is clearly manifested in the similar Au–Au bond lengths according to the different positions of the Au atoms (Fig. S7†). Therefore, Au_38_Cd_4_(DMBT)_30_ can be viewed as the gentle surface reconstruction without breaking the double-helical Au_26_ kernel based on the parent Au_44_(DMBT)_28_. In addition, Au_38_Cd_4_(DMBT)_30_ is also patterned along different directions in the layer structure (Fig. S8†).

To gain an in-depth insight into the Cd-induced surface reconstruction mechanism, density functional theory (DFT) calculations were performed. Starting from the Au_44_(SR)_28_ cluster, as presented in Fig. S9,† four Au_2_(SR)_3_^−^ protecting motifs of Au_44_(SR)_28_ are substituted by six Cd(SR)_2_, resulting in the formation of an unstable intermediate, Au_36_Cd_6_(SR)_28_^4+^, due to the very high substitution energy (24.80 eV, step 1). After this, a surface isomerization of Au_36_Cd_6_(SR)_28_^4+^ is considered *via* the reorganization of the motifs (step 2). In this step, the Cd atom in Cd(SR)_2_ binds with a neighboring S atom in the SR[Au(SR)]_2_^−^ motif, which is accompanied by the breaking of a Au–S bond and the formation of a naked Au atom. The S atom in Cd(SR)_2_ binds with this naked Au atom to form a new Au–S bond. Therefore, the structure in which the three-coordinated μ_3_-Cd atoms bind with two SR^−^ and one Au(SR)_2_^−^ can be obtained during the structural isomerization of Au_36_Cd_6_(SR)_28_^4+^. In addition, the Au_36_Cd_6_(SR)_28_^4+^ becomes more stable after isomerization *via* lowering the energy by 3.37 eV. Then, two S atoms of the SR[Au(SR)]^−^ motif further bind with two μ_3_-Cd atoms to form two four-coordinated μ_4_-Cd atoms, resulting in the formation of a more stable intermediate structure, Au_38_Cd_6_(SR)_32_^2+^, with a formation energy of −18.64 eV (step 3). In step 4, the SR^−^ motif binds with the Cd atom of Cd(SR)_2_ to form Cd(SR)_3_^−^, in which the Cd(SR)_2_ is quickly separated from Cd(SR)_3_^−^ leaving a bridging SR^−^ motif on the surface of the Au core. Finally, the stable Au_38_Cd_4_(SR)_30_ is formed with a formation energy of −12.50 eV. The proposed conversion process from Au_44_(SR)_28_ to Au_38_Cd_4_(SR)_30_ includes two key steps: (i) the substitution of SR[Au(SR)]_2_^−^ by Cd(SR)_2_ and (ii) the structural isomerization of surface ligands.

**Fig. 3:**
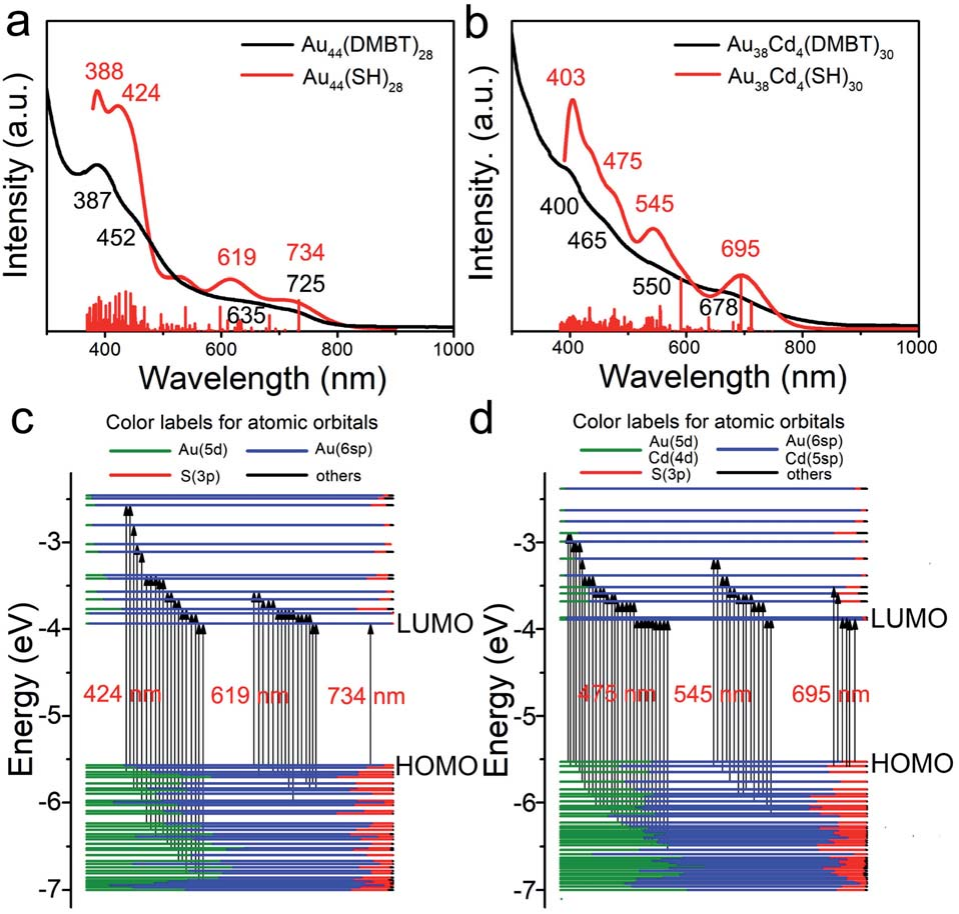
Experimental and calculated UV-vis-NIR spectra of (a) Au_44_(DMBT)_28_ and (b) Au_38_Cd_4_(DMBT)_30_ nanoclusters and molecular orbital (MO) energy level diagrams for (c) Au_44_(SR)_28_ and (d) Au_38_Cd_4_(SR)_30_. The convolution factor of the convoluted spectra is 0.1 eV.

To investigate the electronic structure changes induced by Cd-atom surface modification, the optical adsorption spectra of the Au_44_(DMBT)_28_ and Au_38_Cd_4_(DMBT)_30_ nanoclusters were measured. The absorption peaks of Au_38_Cd_4_(DMBT)_30_ are mainly centered at 400, 465, 550 and 678 nm (Fig. 3b), which differ from those observed in the parent nanocluster (387, 452, 635 and 725 nm; Fig. 3a). These optical features can be well reproduced by theoretical calculations (Fig. 3a, b and S10†). The Kohn–Sham (KS) molecular orbital (MO) energy levels and atomic orbital components in each KS MO of Au_44_(SR)_28_ and Au_38_Cd_4_(SR)_30_ suggest that the absorption peaks mainly involve the Au(sp) → Au(sp) transitions (Fig. 3c and d). In particular, for Au_44_(SR)_28_, the first absorption peak centered at 734 nm originates from the highest occupied molecular orbital → the lowest unoccupied molecular orbital (HOMO → LUMO) transition, while for Au_38_Cd_4_(SR)_30_, the first absorption peak centered at 695 nm originates from the HOMO → LUMO, HOMO → LUMO+1, HOMO → LUMO+4, HOMO−1 → LUMO, HOMO−1 → LUMO+1, and HOMO−1 → LUMO+5 transitions. The more complex orbital transitions in Au_38_Cd_4_(SR)_30_ than in Au_44_(SR)_28_ can be attributed to the dopant Cd. This behaviour can also be observed for other absorption peaks.

**Fig. 4:**
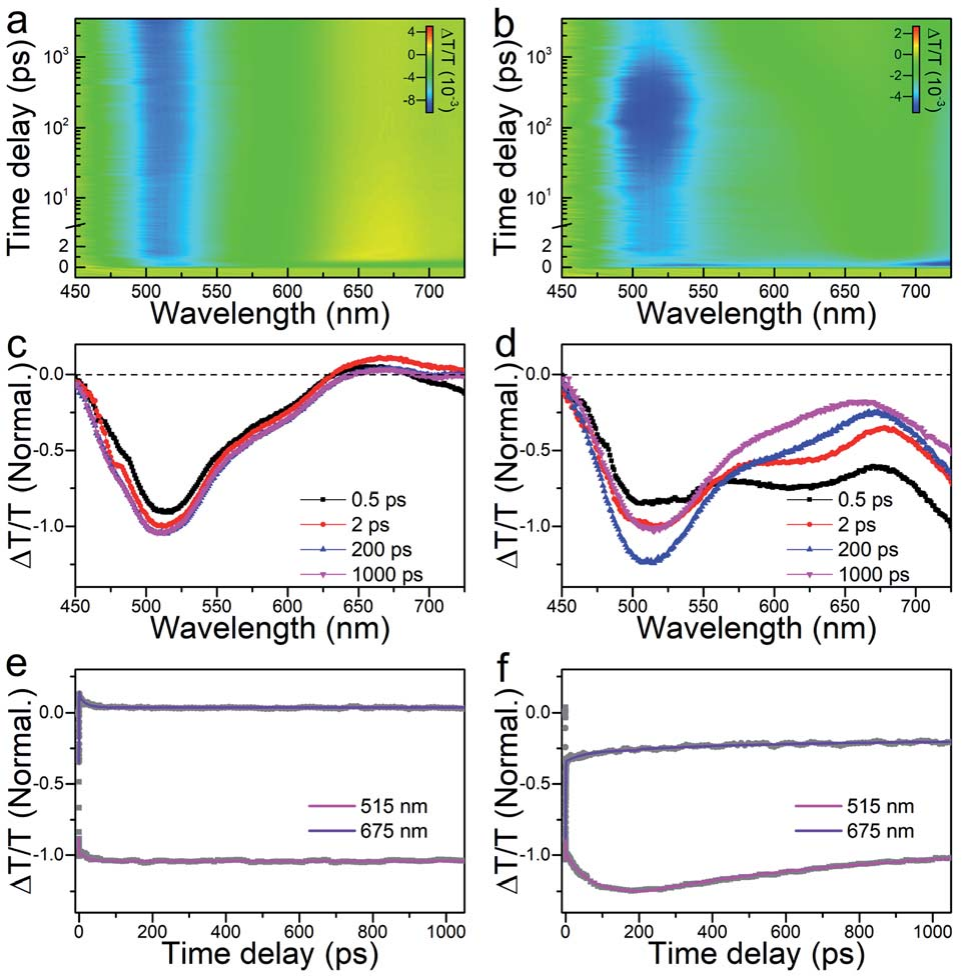
Femtosecond-resolved TA spectra of (a) Au_44_(DMBT)_28_ and (b) Au_38_Cd_4_(DMBT)_30_ pumped at 400 nm. Time evolution of femtosecond-resolved TA spectra of (c) Au_44_(DMBT)_28_ and (d) Au_38_Cd_4_(DMBT)_30_. Kinetic traces at selected wavelengths of (e) Au_44_(DMBT)_28_ and (f) Au_38_Cd_4_(DMBT)_30_. The data are plotted in a scale normalized to the amplitude of the signal probed at 515 nm at a delay of 2 ps. The gray dots in (e) and (f) are the original data, while the corresponding multi-exponential fits are plotted as colored lines.

Moreover, femtosecond and nanosecond carrier dynamics of the two nanoclusters were measured *via* time-resolved transient absorption (TA) spectroscopy to decipher their potential energy-related applications. The femtosecond-resolved TA spectra of the Au_44_(DMBT)_28_ and Au_38_Cd_4_(DMBT)_30_ nanoclusters are provided in Fig. 4a and b. Similarly, both Au_44_(DMBT)_28_ and Au_38_Cd_4_(DMBT)_30_ nanoclusters showed broad excited state absorption (ESA) signals overlapped with ground state bleaching (GSB) peaks near 675 nm. We selectively extracted the TA spectra at different delay times, combined with the dynamic traces probed at 515 and 675 nm to study the transient evolution and the relaxation dynamics (Fig. 4c–f). A 0.6 ps process at the early stage, which is attributed to the ultrafast internal conversion from higher excited states to lower excited states,^29^ was observed in the two nanoclusters (Fig. S11 and Table S3†). It is worth noting that the major divergence between the two nanocluster systems emerged after a delay of 2 ps. For Au_44_(DMBT)_28_, the TA spectra remained nearly unchanged after 2 ps (Fig. 4c), which is consistent with the flat decay kinetic traces shown in Fig. 4e. A 19 ps process obtained by exponential fitting was ascribed to the structural relaxation caused by conformational changes after pumping.^29–31^ For Au_38_Cd_4_(DMBT)_30_, interestingly, an obvious spectral transformation was observed and the lifetime of this component was determined to be 57 ps (Table S3†), which differs from the 19 ps structural relaxation observed in Au_44_(DMBT)_28_ and might be related to the charge transfer states between the ligand and the metal core of Au_38_Cd_4_(DMBT)_30_,^32–35^ which can be manifested by the overall Hirshfeld charge of the Au_26_ core in Au_38_Cd_4_(SH)_30_ (0.46) and in Au_44_(SH)_28_ (0.56). Of note, deduced from nanosecond-resolved TA analysis, as shown in Fig. S12,† the Au_38_Cd_4_(DMBT)_30_ nanocluster exhibits a faster carrier recombination process with a lifetime of 253 ns than the Au_44_(DMBT)_28_ nanocluster (389 ns lifetime), readily supporting the putative synergy in the Au_38_Cd_4_(DMBT)_30_ nanocluster being invoked in underpinning the excited-state dynamics.

**Fig. 5:**
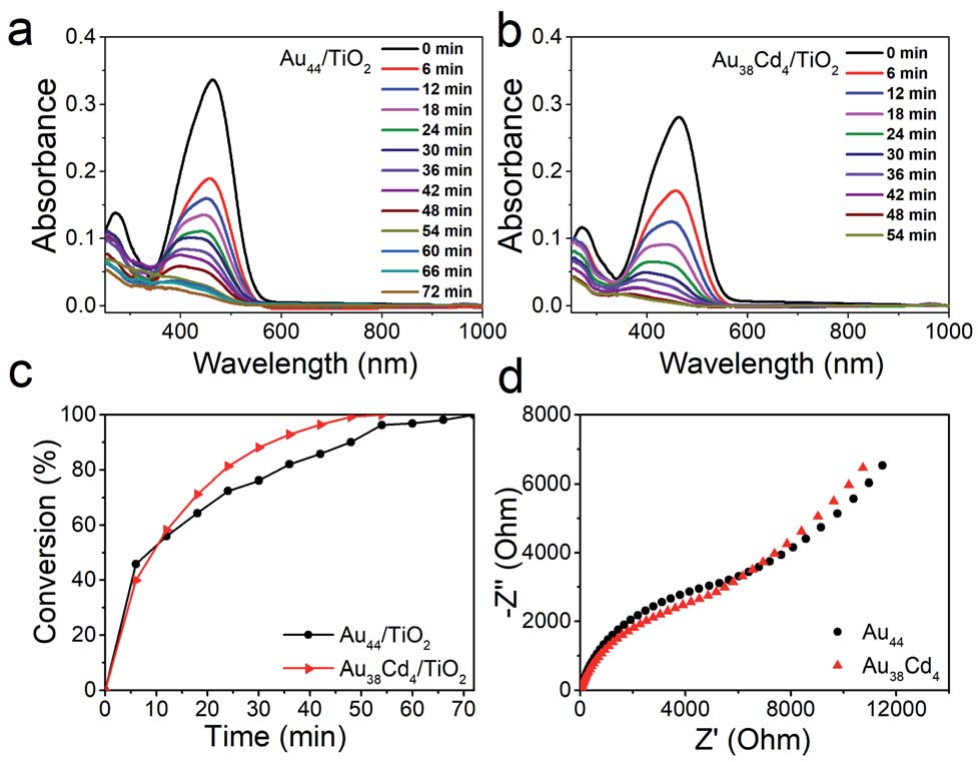
Photocatalytic degradation of methyl orange over (a) Au_44_(DMBT)_28_/TiO_2_ and (b) Au_38_Cd_4_(DMBT)_30_/TiO_2_ under visible light illumination. (c) Visible light-driven degradation of methyl orange by Au_38_Cd_4_(DMBT)_30_/TiO_2_ and Au_44_(DMBT)_28_/TiO_2_ catalysts. Reaction conditions: 40 mg catalyst, 20 mL H_2_O, 0.1 mL (1 g L^−1^) methyl orange. (d) Electrochemical impedance spectra of the Au_38_Cd_4_(DMBT)_30_ and Au_44_(DMBT)_28_ nanoclusters.

The distinguishable electronic and optical properties of the two nanoclusters would apparently impact their catalytic properties. Thus, visible light-driven degradation of methyl orange was selected to explore the photocatalysis of the two nanoclusters. From Fig. 5a and b, within 50 min, methyl orange can be completely degraded on the Au_38_Cd_4_(DMBT)_30_ catalyst under visible light illumination, while on the Au_44_(DMBT)_28_ catalyst it was completed in 70 min. The plots of methyl orange degradation on the catalysts *versus* reaction time further indicate the better photocatalytic performance of the Au_38_Cd_4_(DMBT)_30_ catalyst (Fig. 5c). Electrochemical impedance spectroscopy was performed to investigate the interfacial transfer of electrons. In Fig. 5d, the semicircular diameter of Au_38_Cd_4_(DMBT)_30_ was smaller than that of Au_44_(DMBT)_28_, which implies faster electron-transfer in the Au_38_Cd_4_(DMBT)_30_ system. The photocatalysis difference in the two cluster catalysts is suggested to arise from their different equilibria established between the carrier recombination and the electron transfer influenced by metal synergy.

## Conclusions

In summary, we have developed a Cd-driven surface reconstruction strategy for synthesizing a new Au_38_Cd_4_(DMBT)_30_ bimetallic nanocluster with the fcc Au_26_ core retained from the parent Au_44_(DMBT)_28_ nanocluster. The two nanoclusters that exhibit elegant patterns of Au_4_ tetrahedra show distinct differences in the electronic structures, optical properties, and photocatalytic performances. Beyond the Cd-mediated surface reconstruction case, we anticipate that this heteroatom-doping mechanism will find applications in using gold and other metals in a series of challenging gold-based nanocluster formations and tuning of their intrinsic properties.

## Conflicts of interest

There are no conflicts to declare.

## Supplementary Material

SC-012-D0SC05163B-s001

SC-012-D0SC05163B-s002
