# Supplementary material for: Cd-driven surface reconstruction and photodynamics in gold nanoclusters
Source: Chem Sci. 2021 Jan 5;12(9):3290–4. doi: 10.1039/d0sc05163b (PMC8179392; doi:10.1039/d0sc05163b)
Supplement: SC-012-D0SC05163B-s001 [file SC-012-D0SC05163B-s001.pdf]

Electronic Supplementary Material (ESI) for Chemical Science.
This journal is © The Royal Society of Chemistry 2021

Supplementary Information

Cd-Driven Surface Reconstruction and Photodynamics in Gold Nanoclusters

Xu Liu, Guo Yao, Xinglian Cheng, Jiayu Xu, Xiao Cai, Weigang Hu, Wen Wu Xu, Chunfeng Zhang and Yan Zhu

MATERIALS AND METHODS

Materials

All chemicals and reagents are commercially available and used as received. 3,5-dimethylbenzenethiol (3,5-DMBT, 98%), 4-tert-butylbenzenethiol (TBBT, 98%), chlorotoluene (PhCl, 99%), 4-tert-butyltoluene (95%) and tetra-octylammonium bromide (TOAB, 98%) were purchased from Aladdin. Tetrachloroauric (Ⅲ) acid ($HAuCl_4 \cdot 4H_2O$, 99.9%), sodium borohydride ($NaBH_4$, 98%), methanol (CH_3OH, 99%), dichloromethane (CH_2Cl_2, 99%), acetonitrile (CH_3CN, 99%), toluene ($PhCH_3$, 99%) and petroleum ether (AR) were obtained from Sinopharm Chemical Reagent. Co. Ltd. The water used in all experiments was ultrapure with the resistivity of 18.2 MΩ·cm produced by a Milli-Q NANO pure water system.

Synthesis of $Au_{44}(DMBT)_{28}$ nanoclusters

$Au_{44}(DMBT)_{28}$ nanoclusters were synthesized using a two-step size focusing method. Step 1: 200 mg $HAuCl_4 \cdot 4H_2O$ (0.48 mmol) was dissolved in 2 mL water, which was subsequently mixed with 30 mL CH_2Cl_2 containing tetra-octylammonium

bromide (TOAB, 0.15 mmol). After vigorously stirring for ~30 min, the organic phase was transferred into a 100 mL flask and then 220 μL 3,5-dimethylbenzenethiol (DMBT) was added. This mixture was continuously stirred for 2~3 h until the color of the solution changed clear, and then a freshly prepared ice-water solution with $NaBH_4$ (100 mg) was injected at once. The reaction was continuously to proceed overnight (~10 h). Subsequently, the reaction mixture was dried by a rotary evaporator, and the obtained precipitates were washed with methanol several times to remove excess ligands and salts. Step 2, the precursor was extracted with 2 mL toluene and then etched by 1.5 mL DMBT at 40 °C for 24 h. The crude product was washed with methanol and separated using thin-layer chromatography. $Au_{44}(DMBT)_{28}$ nanoclusters were crystallized in chlorotoluene/methanol solution by vapor diffusion over one week.

Synthesis of $Au_{38}Cd_4(DMBT)_{30}$ nanoclusters

10 mg $Au_{44}(DMBT)_{28}$ nanoclusters were dissolved in toluene (2 mL), which was mixed with 5 mg $Cd(NO_3)_2$ (20 equiv.) dissolved in acetonitrile (1 mL) and 200 μL DMBT ligand. The above mixture was vigorously stirred at 40 °C for 12~14 h. The reaction solution was washed with methanol two times to remove excess ligands. Then the product was separated by thin layer chromatography. Needle-like crystals were obtained by diffusing methanol into 4-tert-butyltoluene solution of $Au_{38}Cd_4(DMBT)_{30}$ over one month.

Characterizations

UV-vis absorption spectra of the nanoclusters were measured on a UV-1800 spectrophotometer (Shimadzu, Japan). ESI-MS spectra were performed on a Waters

QT of mass spectrometer with a Z-spray source. The single crystal X-ray diffraction data for $Au_{44}(DMBT)_{28}$ and $Au_{38}Cd_4(DMBT)_{30}$ nanoclusters were collected on a Bruker D8 VENTURE using Mo Kα radiation (λ = 0.71073 Å). The electrochemical impedance spectroscopy (EIS) measurement was performed on a CHI660B electrochemical workstation. A conventional three-electrode system was employed for the experiments. A platinum foil and a saturated Ag/AgCl electrode were used as counter electrode (CE) and reference electrode (RE), respectively. Working electrode (WE) was a glassy carbon. All the experiments were carried out in 1 mol/L Na_2SO_4 at room temperature.

Ultrafast optical measurements

Femtosecond-resolved TA spectroscopy was conducted using a Ti:sapphire regenerative amplifier (Libra, Coherent Inc.; 800 nm, ~100 fs, 1 kHz). A portion of the laser was used to generate the pump beam at 400 nm by the second harmonic generation on BBO crystal. Another small portion of the laser was focused into a 3 mm-thick sapphire plate to produce a broadband supercontinuum light in the visible region. The pump and probe beams were overlapped in time and space in the sample with their optical delay controlled by a translation stage. The TA signal was then detected by a high-speed charge-coupled device (CCD; S11071-1104, Hamamatsu) with a monochromator (Acton 2358, Princeton Instrument) at 1 kHz enabled by a custom-built board from Entwicklungsbuero Stresing. The signal to noise ratio in differential transmission was better than 5×10^{-5} after accumulating and averaging 1,000 pump-on and pump-off shots for each data point. Nanosecond-resolved TA measurements were

performed using the same supercontinuum white light as the probe beam, while the pump beam at 355 nm was adopted by the triple frequency generation of a q-switched Nd:YVO4 laser with ~800 ps pulse width (Picolo AOT MOPA, InnoLas Laser). The synchronization and temporal delay were achieved using an electronic delay generator (SRS DG645, Stanford Research System). The polarization between the pump and probe pulse was set to magic angle (54.7°) for both experiments. The pump fluence was kept about 80 $\mu J/cm^2$ and 150 $\mu J/cm^2$ in fs and ns experiments, respectively. The nanoclusters were dissolved in toluene and placed in 1 mm path length cuvettes for TA measurements.

Photocatalytic reaction

40 mg catalyst (2 wt% cluster) was dispersed in 20 mL H_2O under stirring. Then 0.1 mL 1 g/L methyl orange was added and the solution was irradiated with the visible light using a Xe lamp by filtering the light wavelength < 400 nm. Samples from the solution were taken every 6 minutes and measured with UV-vis spectrometer.

Computational Details

All of the structural optimizations in this study were performed using density functional theory (DFT) method implemented in the Gaussian 09 program package with the Perdew-Burke-Ernzerhof (PBE) functional.[S1,2] The all-electron basis set 6-31G* for H and S and the effective-core basis set LANL2DZ for Au were adopted. The time-dependent DFT calculations were applied to simulate the absorption spectra. The DMBT groups were replaced by SH groups to lower the computational cost.

References

1. M. J. Frisch, G. W. Trucks, H. B. Schlegel, G. E. Scuseria, M. A. Robb, J. R.

Cheeseman, G. Scalmani, V. Barone, B. Mennucci, G. A. Petersson, H. Nakatsuji, M. Caricato, X. Li, H. P. Hratchian, A. F. Izmaylov, J. Bloino, G. Zheng, J. L. Sonnenberg, M. Hada, M. Ehara, K. Toyota, R. Fukuda, J. Hasegawa, M. Ishida, T. Nakajima, Y. Honda, O. Kitao, H. Nakai, T. Vreven, J. J. A. Montgomery, J. E. Peralta, F. Ogliaro, M. Bearpark, J. J. Heyd, E. Brothers, K. N. Kudin, V. N. Staroverov, R. Kobayashi, J. Normand, K. Raghavachari, A. Rendell, J. C. Burant, S. S. Iyengar, J. Tomasi, M. Cossi, N. Rega, N. J. Millam, M. Klene, J. E. Knox, J. B. Cross, V. Bakken, C. Adamo, J. Jaramillo, R. Gomperts, R. E. Stratmann, O. Yazyev, A. J. Austin, R. Cammi, C. Pomelli, J. W. Ochterski, R. L. Martin, K. Morokuma, V. G. Zakrzewski, G. A. Voth, P. Salvador, J. J. Dannenberg, S. Dapprich, A. D. Daniels, Ö. Farkas, J. B. Foresman, J. V. Ortiz, J. Cioslowski and D. J. Fox, Gaussian 09, Revision B.1, Gaussian, Inc., Wallingford CT, 2009.

2. J. P. Perdew, K. Burke and M. Ernzerhof, *Phys. Rev. Lett*. 1996, **77**, 3865-3868.

Supporting Figures and Tables

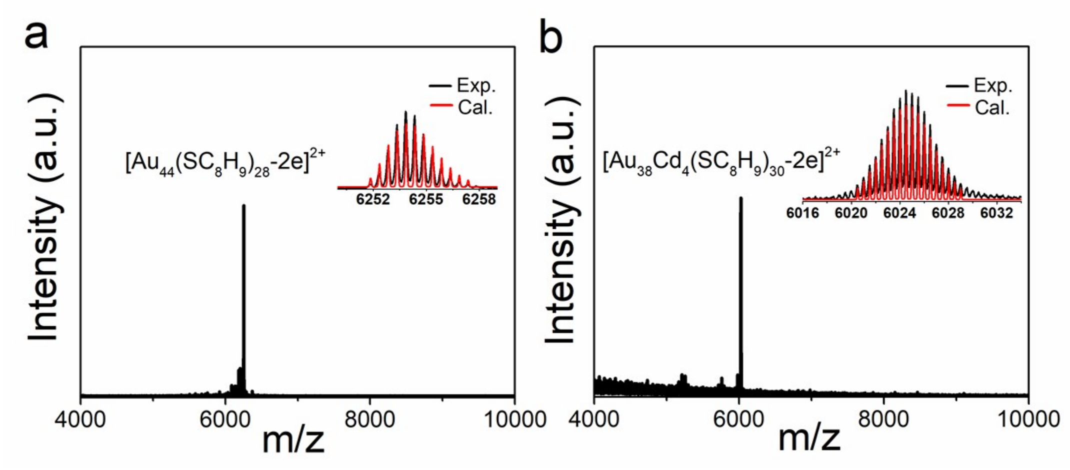

Fig. S1. ESI-MS spectra of (a) $Au_{44}(DMBT)_{28}$ and (b) $Au_{38}Cd_4(DMBT)_{30}$ nanoclusters. Insets are experimental and calculated isotopic patterns.

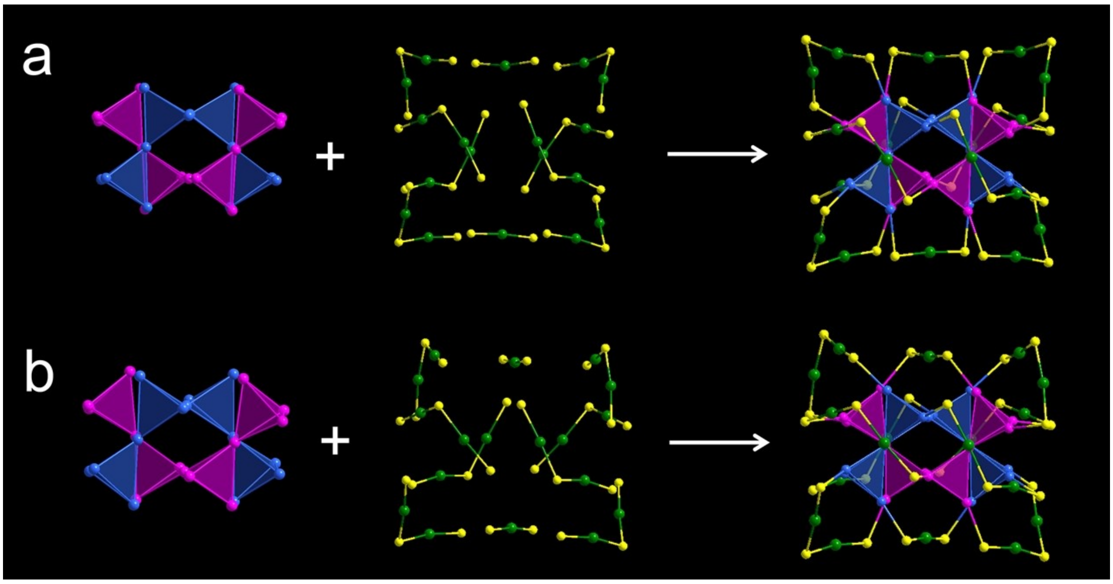

Fig. S2. Structural comparison between (a) $Au_{44}(TBBT)_{28}$ and (b) $Au_{44}(DMBT)_{28}$ nanoclusters. Color codes: blue/magenta/green = Au, yellow = S. C and H atoms are omitted for clarity.

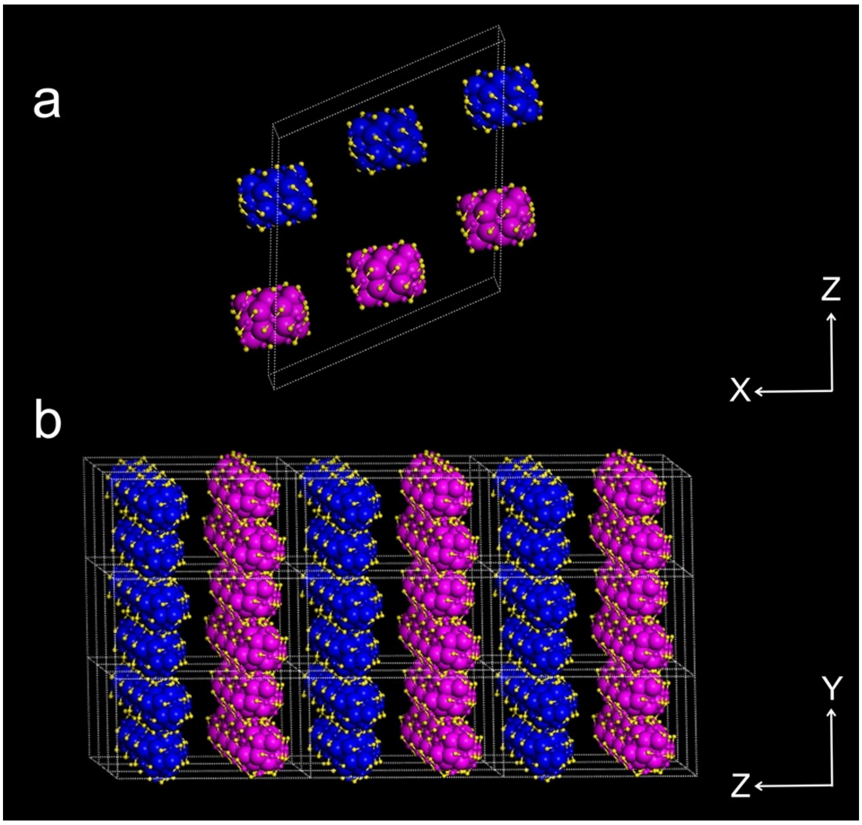

Fig. S3. The packing mode of $Au_{44}(TBBT)_{28}$ nanoclusters. (a) Parallel hexahedron unit cell of $Au_{44}(TBBT)_{28}$ with three pairs of enantiomers viewed from Y axis. (b) Packing structure of $Au_{44}(TBBT)_{28}$ nanoclusters along X axis. The left-hand and right-handed enantiomers are shown in the magenta and blue colors, respectively. Color codes: blue/magenta = Au, yellow = S. C and H atoms are omitted for clarity.

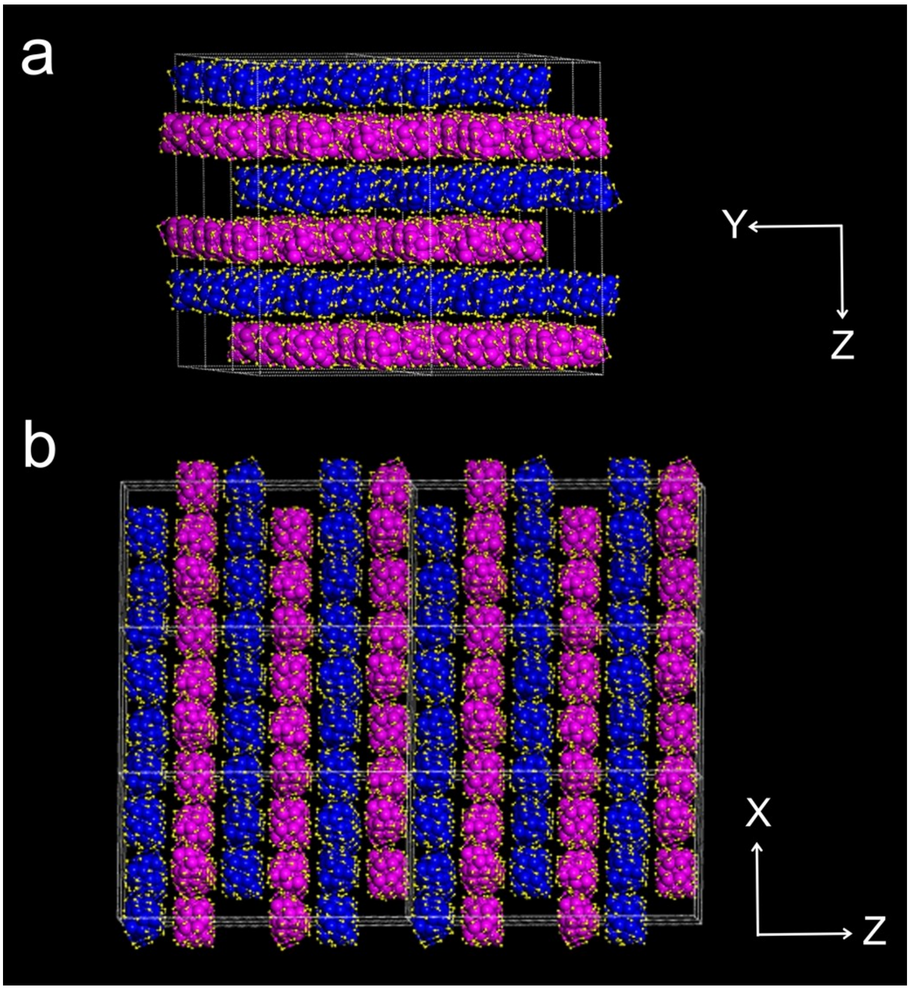

Fig. S4. The packing mode of $Au_{44}(DMBT)_{28}$ nanoclusters. Packing structures of $Au_{44}(DMBT)_{28}$ nanoclusters along (a) X axis and (b) Y axis, respectively. The left-hand and right-handed enantiomers are shown in the magenta and blue colors, respectively. Color codes: blue/magenta = Au, yellow = S. C and H atoms are omitted for clarity.

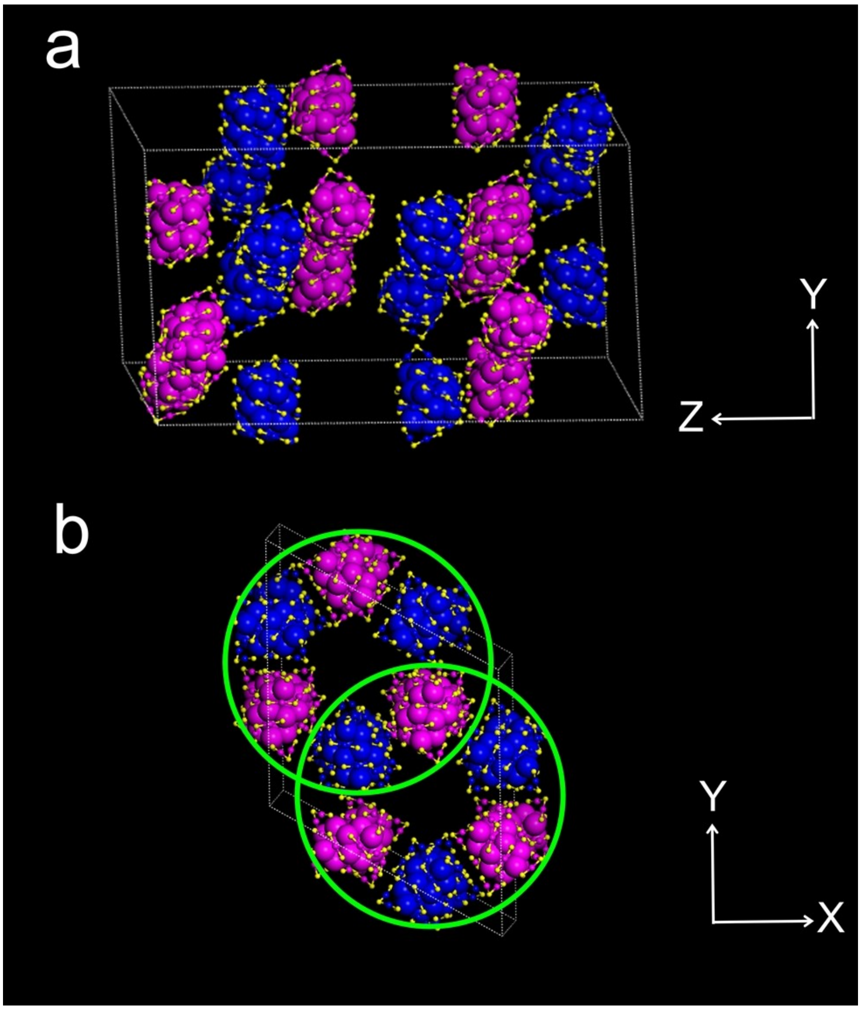

Fig. S5. The packing mode of $Au_{44}(DMBT)_{28}$ nanoclusters in the unit cell. Parallel hexahedron unit cell of $Au_{44}(DMBT)_{28}$ with some pairs of enantiomers viewed from (a) X axis and (b) Z axis, respectively. Color codes: blue/magenta = Au, yellow = S. C and H atoms are omitted for clarity.

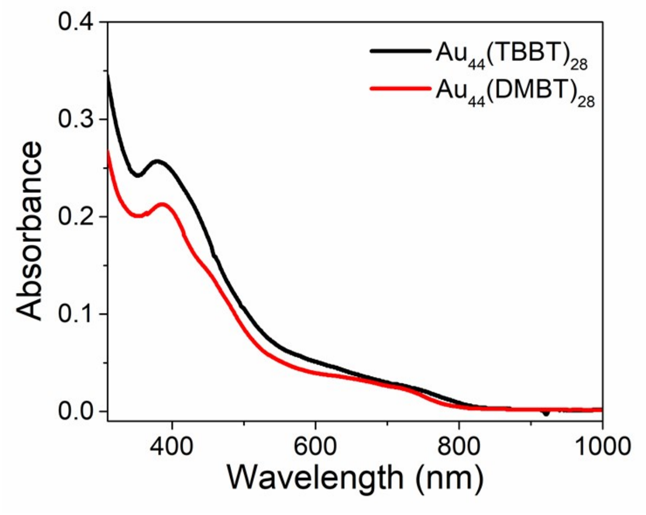

Fig. S6. UV-vis-NIR absorption spectra of $Au_{44}(TBBT)_{28}$ and $Au_{44}(DMBT)_{28}$ nanoclusters.

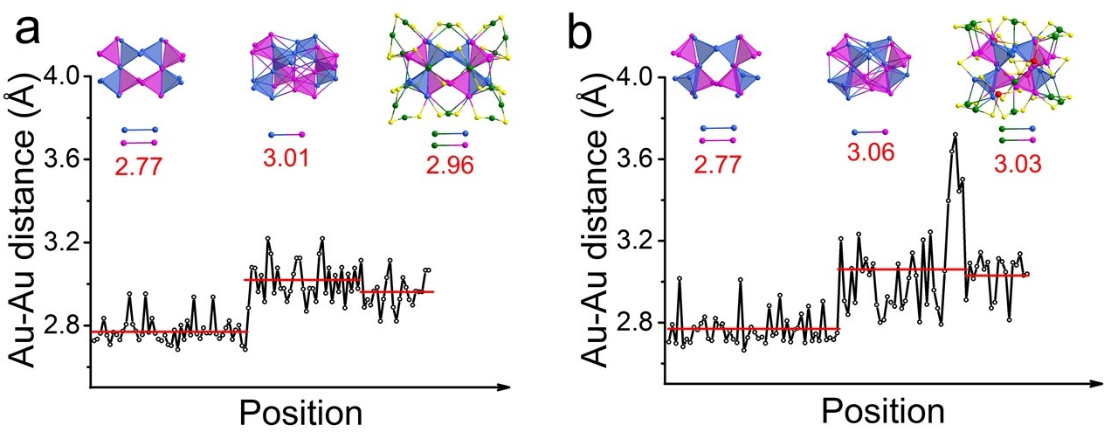

Fig. S7. Distribution of Au-Au bond lengths from the different positions of the Au atoms of (a) $Au_{44}(DMBT)_{28}$ and (b) $Au_{38}Cd_4(DMBT)_{30}$ nanoclusters.

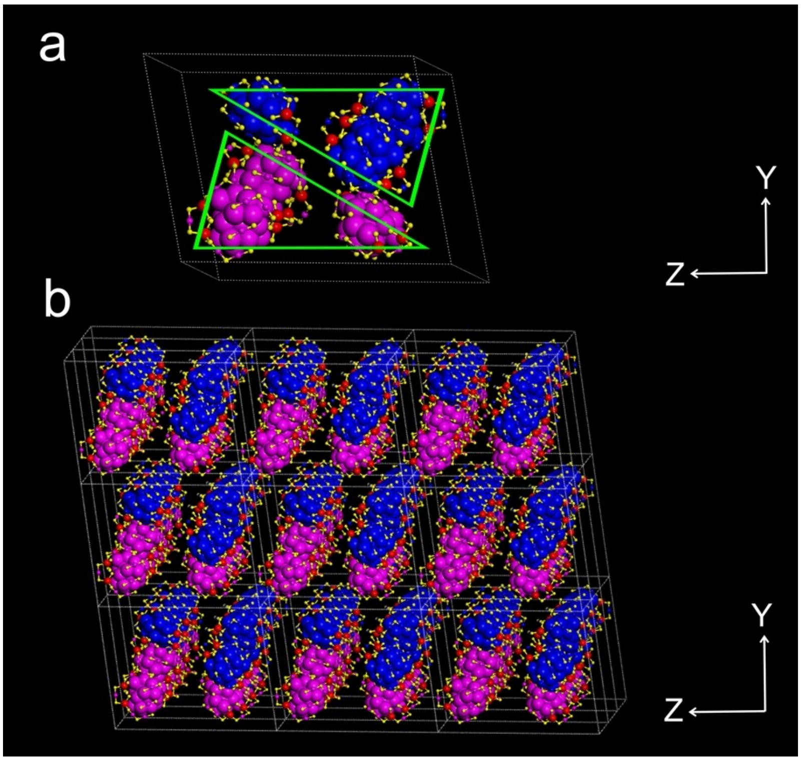

Fig. S8. The packing mode of $Au_{38}Cd_4(DMBT)_{30}$ nanoclusters. (a) Parallel hexahedron unit cell of $Au_{38}Cd_4(DMBT)_{30}$ with three pairs of enantiomers viewed from X axis. (b) Packing structure of $Au_{38}Cd_4(DMBT)_{30}$ nanoclusters along X axis. The left-hand and right-handed enantiomers are shown in the magenta and blue colors, respectively. Color codes: blue/magenta = Au, red = Cd, yellow = S. C and H atoms are omitted for clarity.

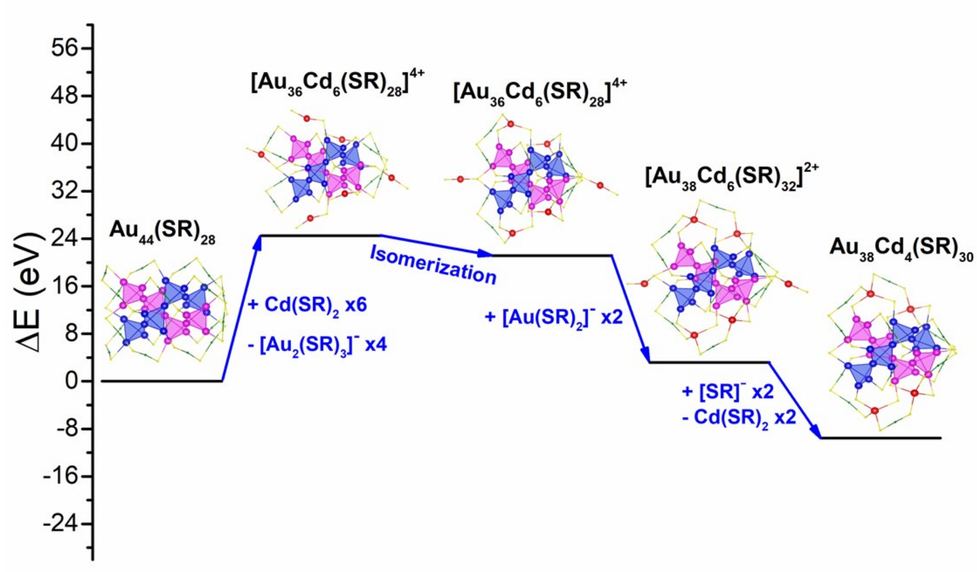

Fig. S9 Theoretically proposed evolution mechanism of $Au_{38}Cd_4(SR)_{30}$ from $Au_{44}(SR)_{28}$ triggered by Cd-SR complex. Note that $Au_{44}(SR)_{28}$ is referenced for the energy. Color codes: blue/magenta/green = Au, yellow = S, red = Cd.

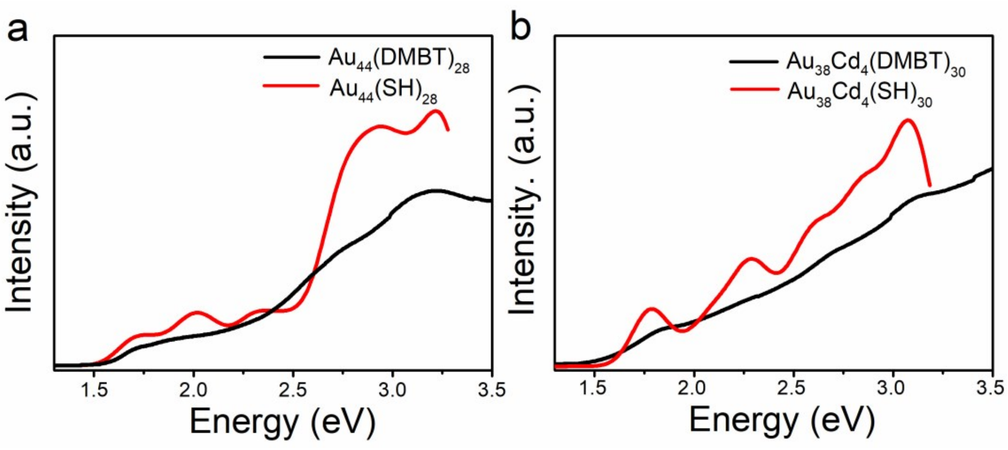

Fig. S10 Experimental and calculated UV-vis-NIR spectra of (a) $Au_{44}(DMBT)_{28}$ and (b) $Au_{38}Cd_4(DMBT)_{30}$ nanoclusters on energy scale.

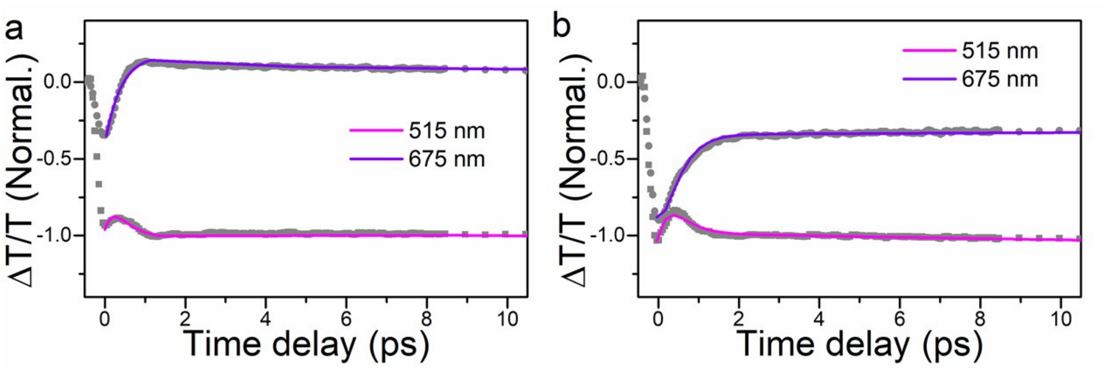

Fig. S11. Selected kinetic traces and the corresponding exponential fits of (a) $Au_{44}(DMBT)_{28}$ and (b) $Au_{38}Cd_4(DMBT)_{30}$ with femtosecond resolution.

Fig. S12. Selected kinetic traces and the corresponding exponential fits of (a) $Au_{44}(DMBT)_{28}$ and (b) $Au_{38}Cd_4(DMBT)_{30}$ with nanosecond resolution.

Table S1. Crystal data and structure refinement for the $Au_{44}(DMBT)_{28}$ nanocluster.

Empirical formula	C224 H252 Au44S28	
Formula weight	12508.45	
Temperature	193 K	
Wavelength	0.71073 Å	
Crystal system	Trigonal	
Space group	R -3c	
Unit cell dimensions	a = 45.692(2) Å	α = 90°
	b = 45.692(2) Å	β = 90°
	c = 77.435(4) Å	γ = 120°
Volume	140008(14) $Å^3$	
Z	18	
Absorption coefficient	20.874 mm^{-1}	
F(000)	99360	
Theta range for data collection	1.929 to 27.481°	
Index ranges	-59<=h<=59, -59<=k<=52, -96<=l<=100	
Reflections collected	432161	
Independent reflections	35644 [R(int) = 0.1146]	
Completeness to theta = 25.242°	99.9 %	
Absorption correction	Semi-empirical from equivalents	
Max. and min. transmission	0.2616 and 0.0856	
Refinement method	Full-matrix least-squares on F^2	
Data / restraints / parameters	35644 / 1789 / 1351	
Goodness-of-fit on F^2	1.016	
Final R indices [I>2sigma(I)]	R1 = 0.0488, wR2 = 0.1309	
R indices (all data)	R1 = 0.0923, wR2 = 0.1596	
Largest diff. peak and hole	3.424 and -2.632 $eÅ^{-3}$	

Table S2. Crystal data and structure refinement for the $Au_{38}Cd_4(DMBT)_{30}$ nanocluster.

Empirical formula	C240 H270 Au38Cd4S30	
Formula weight	12050.67	
Temperature	193 K	
Wavelength	0.71073 Å	
Crystal system	Triclinic	
Space group	P-1	
Unit cell dimensions	a = 35.243(3) Å	α = 78.898(3)°
	b = 36.193(3) Å	β = 74.680(3)°
	c = 46.321(4) Å	γ = 79.183(3)°
Volume	55331(8) $Å^3$	
Z	6	
Absorption coefficient	15.470 mm^{-1}	
F(000)	32304	
Theta range for data collection	1.933 to 25.290°	
Index ranges	-42<=h<=42, -43<=k<=40, -55<=l<=55	
Reflections collected	432873	
Independent reflections	199500 [R(int) = 0.0661]	
Completeness to theta = 25.242°	99.3 %	
Absorption correction	Semi-empirical from equivalents	
Max. and min. transmission	0.0962 and 0.0310	
Refinement method	Full-matrix least-squares on F^2	
Data / restraints / parameters	199500 / 109786 / 9099	
Goodness-of-fit on F^2	0.953	
Final R indices [I>2sigma(I)]	R1 = 0.0551, wR2 = 0.1486	
R indices (all data)	R1 = 0.1086, wR2 = 0.1750	
Largest diff. peak and hole	2.907 and -2.697 $eÅ^{-3}$	

Table S3. Time constants obtained by multi-exponential fit of the selected kinetic traces from femto/nanosecond-resolved TA spectroscopic measurements.

Sample	Decay time constants		
$Au_{44}(DMBT)_{28}$	0.6 ps ± 0.1 ps	19 ps ± 0.5 ps	389 ns ± 10 ns
$Au_{38}Cd_4(DMBT)_{30}$	0.6 ps ± 0.1 ps	57 ps ± 0.5 ps	10 ns ± 1 ns (rel. amplitude: ~33%)<br>253 ns ± 10 ns (rel. amplitude: ~67%)
